# MiR-30a targets IL-1α and regulates islet functions as an inflammation buffer and response factor

**DOI:** 10.1038/s41598-017-05560-1

**Published:** 2017-07-13

**Authors:** Xin Jiang, Chenke Xu, Fan Lei, Meijian Liao, Wei Wang, Naihan Xu, Yaou Zhang, Weidong Xie

**Affiliations:** 1 School of Life Sciences, Tsinghua University, Beijing, 100084 China; 2 Shenzhen Key Lab of Health Science and Technology, Division of Life Science & Health, Graduate School at Shenzhen, Tsinghua University, Shenzhen, 518055 China; 3 Laboratory of Pharmaceutical Science, School of Life Science, School of Medicine, Tsinghua University, Beijing, 100084 China; 4 Open FIESTA Center, Tsinghua University, Shenzhen, 518055 China

## Abstract

Diabetes is an inflammatory disease. Inflammation plays an important role in islet functions. However, the exact mechanisms by which inflammation affects islet functions remain unclear. In this study, we investigated the regulatory effects of miR-30a on inflammation and islet functions. The results indicate that miR-30a serves as an inflammation-resolving buffer factor by targeting interleukin 1a (IL-1α) in immune cells and in islet cells, which might play an important role in inflammation homeostasis. miR-30a ameliorates islet functions in an inflammatory micro-environment by targeting the IL-1α/nuclear factor kappa B (NFKB) p65 subunit (p65)/p62 (SQSTM1)/insulin axis, which can be developed into a novel antidiabetic approach. miR-30a serves as a promising inflammation-response biomarker in inflammatory diseases and is possibly activated by the toll-like receptor 4 (TLR4)/IL-1α/NFKB pathways. However, the exact molecular mechanisms by which miR-30a regulates inflammation and islet functions as well as the potential applications in transitional medicine require further elucidation.

## Introduction

Diabetes globally has high morbidity and mortality, and it severely threatens human health. Islet dysfunction is a prime effector of the pathogenesis of diabetes^1^. Although numerous new drugs have ideal hypoglycemic effects^2^, there is not an ideal drug to normalize the dysfunction of islets. Diabetes is an inflammatory disease^3^, and inflammation plays an important role in islet impairment and diabetes development^4^. The micro-environment of inflammation in islets is created by the release of cytokines from migrant immune cells and production of cytokines from the islets. Despite limited human data, *in vitro* and preclinical studies have indicated that cytokines released from inflammatory immune cells serve as the prime effector of inflammatory β-cell impairment^5^. Moreover, inflammatory stress in islet cells stimulates the production of cytokines and contributes to β-cell impairment.

Interleukin 1 (IL-1) is an important inflammatory mediator family that causes islet dysfunctions and induces diabetes pathogenesis^6, 7^. IL-1α and IL-1β are the main subtypes of the IL-1 family^8^. IL-1α has similar effects to IL-1β by binding to the IL-1 receptor 1 (IL-1R). Excess IL-1α can activate the NFKB pathway^9^, increase oxidative stress^10^, and promote apoptosis and death^11^ in cells subjected to inflammatory stimulation. Although both IL-1α and IL-1β can interact with IL-1R1 to induce downstream transcription of inflammatory genes, they show considerable differences in their localization, regulation, and function. The IL-1α precursor is constitutively expressed in all cell types. The IL-1α precursor can directly act as a biologically active form^12^. Apart from its release from necrotic cells, IL-1α can translocate to the nucleus after activation by endotoxin and promote the expression of pro-inflammatory transcription factors^13, 14^. Although IL-1R1 is blocked, intracellular overexpression of the IL-1a precursor is sufficient to activate NFKB and activator protein 1 (AP-1).

Intracellular IL-1α is an important target of mediated inflammation. Islet cells possibly produce IL-1α, which causes direct islet cells damage and might explain why extracellular IL-1β antibody (canakinumab) or IL-1R antagonist (anakinra)^15^ does not work well in the clinical setting. Therefore, the production and release of IL-1α in inflammatory cells or pancreatic islets should be inhibited. However, molecules that can control intracellular IL-1α production remain unidentified.

MiRNAs are small non-coding RNAs that are 18–22 nucleoids long. MiRNAs inhibit the translation or degradation of mRNAs by binding to the 3′-untranslated region (3′-UTR). Apart from cell differentiation, development, and insulin secretion, miRNAs are also involved in regulating inflammation in pancreatic islets^16^. Mmu-miR-30a-5p (miR-30a) is predicted to bind to the 3′-UTR of Il-1a mRNA by bioinformatics software (http://www.targetscan.org), which shows a good conserved site in many species, including mice and humans. Considering the possible role of IL-1α in islet inflammation and functions, we speculated that miR-30a regulates inflammation and islet functions. Previous studies demonstrated that miR-30a mainly mediates cell growth^17^, ischemia-induced cell death^18^, autophagy^19^, immune inflammation^20^, and the endoplasmic reticulum^21^. However, whether miR-30a regulates islet inflammation remains unclear. Therefore, the present study investigated whether miR-30a regulates immune and islet cell inflammation and function by targeting IL-1α and found that 30a serves as a promising buffer and response factor in inflammatory diseases including diabetes.

## Results

### MiR-30a binding to the Il-1a-3′-UTR

**Figure 1:**
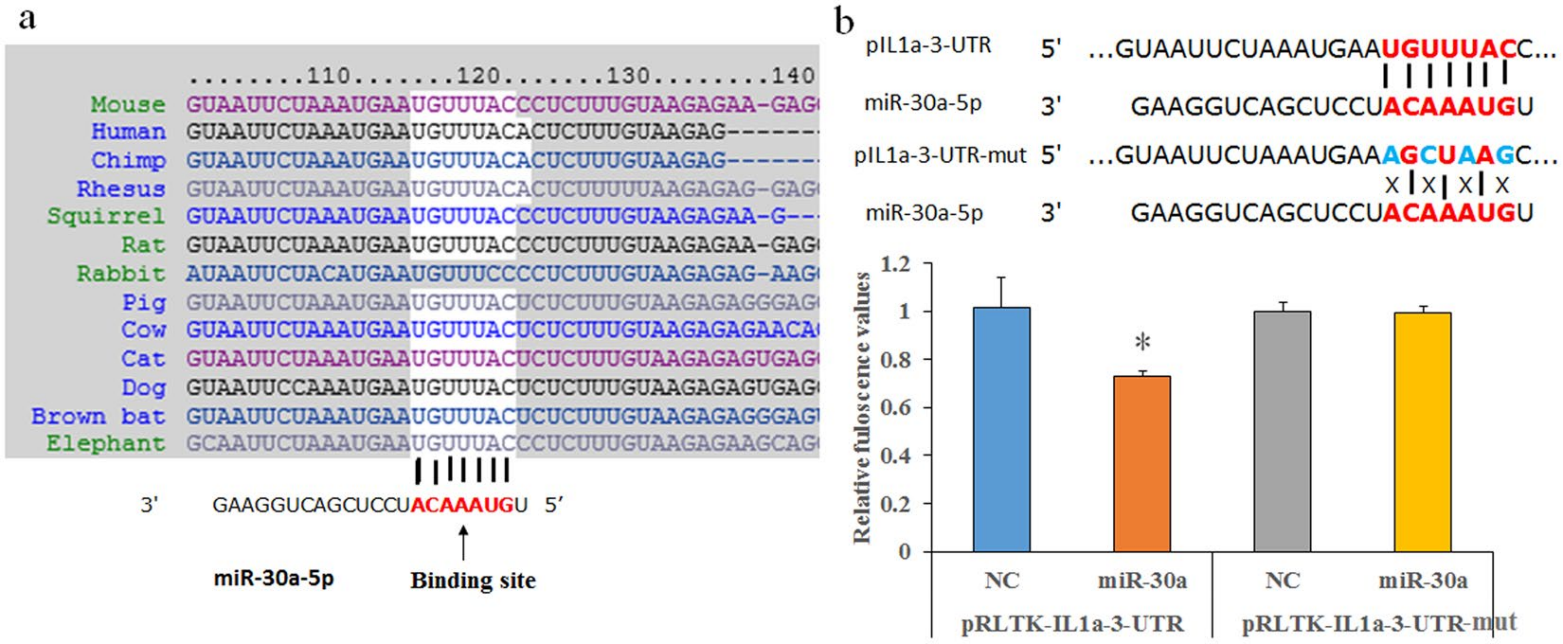
Target prediction and validation of miR-30a and Il-1a-3-UTR. (**a**) Targetscan software showed that miR-30a has a conserved binding site in the Il-1a-3′-UTR in many species including mice and human. (**b**) Luciferase reporter assay showing miR-30a mimics significantly inhibited the fluorescence values in the pRL-TK-Il-1a-3′-UTR-transfected 293T cells compared to negative controls, which was not the case in the mutated vector (pRL-TK-Il-1a-3′-UTR-mut)-transfected 293T cells. NC, negative controls of miRNA mimics; data are expressed as the Mean ± SD (n = 3); *P < 0.05 vs NC.

The binding sequence between the Il-1a-3′-UTR and miR-30a was predicted using Targetscan software, and it was found to be highly conserved in mice, humans, and other species (Fig. 1a). Subsequently, the Il-1a-3′-UTR fragment containing a binding site sequence for miR-30a and its binding site mutant variants were successfully cloned into pRLTK vectors as described below (see the methods section). Then, the vectors and miR-30a mimics were co-transfected into 293T cells. After 24 h of co-transfection, we conducted luciferase reporter assays and found that miR-30a mimics significantly inhibited the chemiluminescence of 293T cells that were co-transfected with pRL-TK-Il-1a-3′-UTR compared to negative controls (Fig. 1b). However, miR-30a mimics had no effect on the chemiluminescence of 293T cells transfected with the vector that had mutated fragments. These results validate that miR-30a specifically binds to the predicted site of the Il-1a-3′-UTR.

### Effects of miR-30a on cytokines in RAW264.7 and Beta-TC-6 cells with inflammatory stimulation

We tested whether miR-30a exerts regulatory effects on IL-1α and other inflammatory factors [interleukin 6 (IL-6) and tumor necrosis factor alpha (TNF-α)] at the transcriptional and translational levels in RAW264.7 (mouse macrophage cell line) and Beta-TC-6 (mouse pancreas beta cell line) cells subjected to inflammatory stimulation. Here, lipopolysaccharides (LPS) at a final concentration of 2 µg/mL, was used to stimulate the inflammation in RAW264.7 cells. However, unlike RAW264.7 cells, Beta-TC-6 cells were insensitive to LPS stimulation alone. We collected a mixture of LPS (2 µg/mL, 12 h of incubation)-induced RAW264.7 cell medium (LRM) and a common medium (1:3, v/v) to create an ideal inflammatory model in Beta-TC-6 cells. The addition of LRM for 12 h significantly increased the expression levels of Il-1a, Il-6 and TNF-a in Beta-TC-6 cells, while LPS did not work as well in Beta-TC-6 as it did in RAW264.7 cells in the preliminary experiments (Supplementary Fig. S1). Hence, we selected LRM as the inflammatory stimulators for Beta-TC-6 cells in the subsequent experiments.

**Figure 2:**
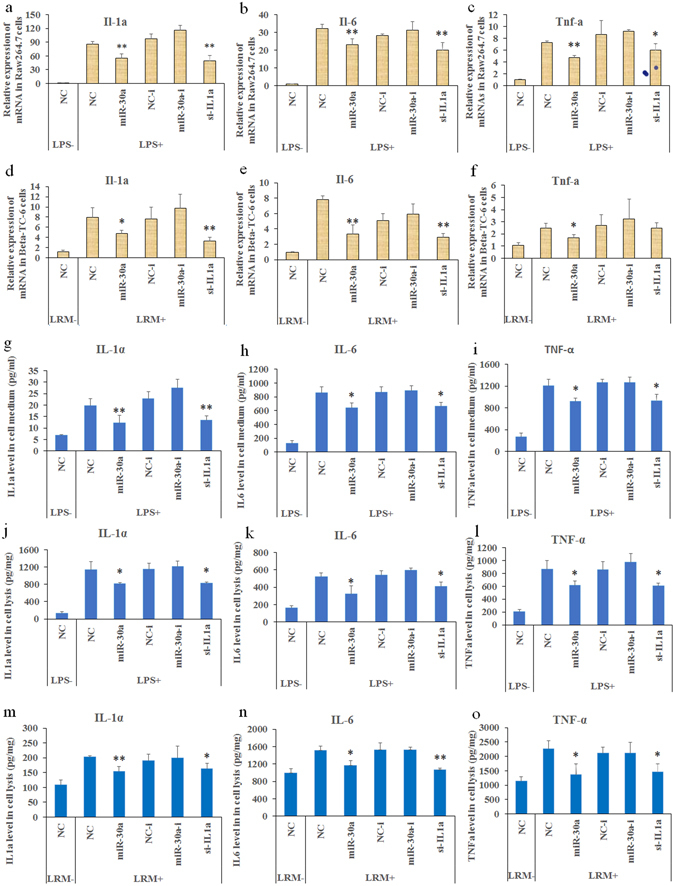
Effects of miR-30a mimics on the mRNA and protein expressions of inflammatory factors. (**a**–**f**) RT-PCR showing miR-30a mimics significantly inhibited the increased mRNA expressions of Il-1a, Il-6 and TNF-a in LPS-induced RAW264.7 and LRM-induced Beta-TC-6 cells. (**g**–**o**) ELISA showing that miR-30a mimics significantly inhibit the increased protein levels of IL-1α, IL-6 and TNF-α in RAW264.7 cells after 24 h of LPS induction (samples from cell medium and lysis) as well as in Beta-TC-6 cells after 24 h of LRM induction (samples from cell lysis only). LPS+/−, incubation with or without LPS; LRM+/−, incubation with or without LRM; NC, negative controls of miRNA mimics; NC-i, negative controls of miRNA inhibitors or siRNA; miR-30a-i, miRNA-30a inhibitor; and si-IL1a, silencing mRNA fragment of Il-1a. Data are expressed as the Mean ± SD (n = 3); *P < 0.05 and **P < 0.01 vs. NC or NC-i (LPS/LRM+), respectively.

RT-PCR results showed that miR-30a mimics significantly inhibited the increased levels of Il-1a, Il-6 and TNF-a mRNAs in both LPS-stimulated RAW264.7 cells and LRM-induced Beta-TC-6 cells (Fig. 2a–f). Si-Il-1a also exhibited similar effects to miR-30a mimics, except for TNF-a expression in Beta-TC-6 cells. However, miR-30a inhibitors did not have significant effect.

For protein expression, enzyme linked immunosorbent assay (ELISA) show that miR-30a significantly inhibited the increased levels of IL-1α, IL-6, and TNF-α in both cell medium and cell lysis of LPS-induced RAW264.7 cells compared with the negative controls (Fig. 2g–l). However, differentiating between the existing and released inflammatory factors in the medium of the LRM-induced Beta-TC-6 cells was difficult. In addition, there were relatively few released inflammatory factors from Beta-TC-6 cells, and they were difficult to assay in the medium. Therefore, we only detected the levels in the cell lysis of the LRM-induced Beta-TC-6 cells. In the LRM-induced Beta-TC-6 cells, miR-30a significantly inhibited the increase of the IL-1α, IL-6, and TNF-α levels in the cell lysis conditions compared with the negative controls (Fig. 2m–o). Si-Il-1a showed comparable effects to miR-30a. However, miR-30a inhibitors did not have any significant effect.

### Effects of miR-30a on reactive oxygen species (ROS) in RAW264.7 and Beta-TC-6 cells with inflammatory stimulation

We assayed the oxidative stress induced by inflammatory stimulation. Produced intracellular ROS can bind to 2,7-Dichlorodi-hydrofluorescein (DCFH) and emit fluorescence.Through fluorescence microscopy, we found that miR-30a significantly inhibited the increased production of ROS in both LPS-induced RAW264.7 and LRM-induced Beta-TC-6 cells compared to the negative controls (Supplementary Fig. S2). Si-Il-1a also exhibited similar effects to miR-30a. However, miR-30a inhibitors did not have any significant effect.

### Effects of miR-30a on apoptosis in Raw264.7 and Beta-TC-6 cells with inflammatory stimulation

**Figure 3:**
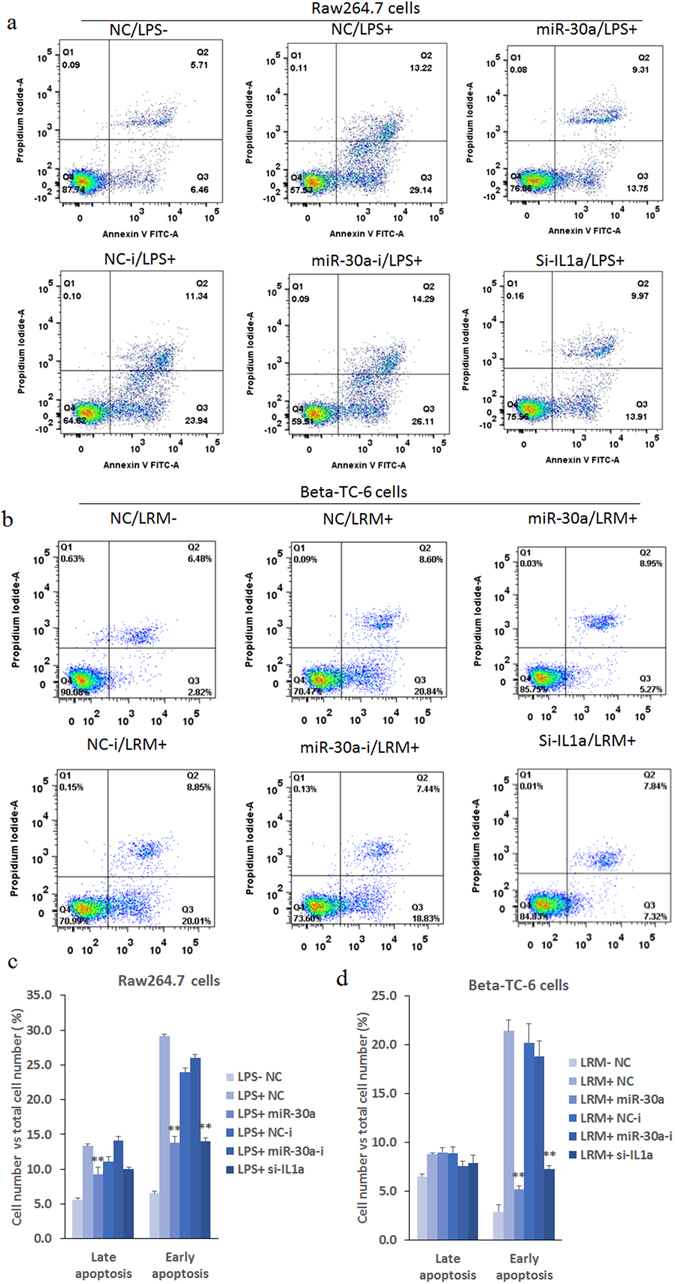
Effects of miR-30a mimics on apoptosis in RAW264.7 and Beta-TC-6 cells subjected to inflammatory stimulation measured by flow cytometry. (**a** and **c**) Flow cytometry showing that miR-30a mimics significantly inhibit the increased apoptosis in LPS-induced RAW264.7 cells. (**b** and **d**) Flow cytometry showing that miR-30a mimics significantly inhibited the increased apoptosis in LRM-induced Beta-TC-6 cells. LPS+/−, incubation with or without LPS; LRM+/−, incubation with or without LRM; NC, negative controls of miRNA mimics; NC-i, negative controls of miRNA inhibitors or siRNA; miR-30a-i, miRNA-30a inhibitor; and si-IL1a, silencing mRNA fragment of Il-1a. Data are expressed as the Mean ± SD (n = 3); **P < 0.01 vs. NC or NC-i (LPS/LRM+), respectively.

We also assayed the early and late apoptosis in LPS-induced RAW264.7 and LRM-induced Beta-TC-6 cells by flow cytometry. The cells in the state of early apoptosis can only be labeled with Annexin V, while cells in late apoptosis can be associated with necrotic cell death and be labeled with both Annexin V and propidium iodide (PI). MiR-30a significantly inhibited the increase in early apoptosis (Q3) in both RAW264.7 and Beta-TC-6 cells induced by 24 h of LPS and LRM incubation, respectively, which was comparable to si-Il-1a (Fig. 3a–d). MiR-30a exerted a significant effect on late apoptosis (Q2) in RAW264.7 cells; however, MiR-30a did not induce significant effects on Beta-TC-6 cells. Additionally, miR-30a inhibitors did not have any significant effects in RAW264.7 or Beta-TC-6 cells.

### Effects of miR-30a on insulin levels in Beta-TC-6 cells with inflammatory stimulation

**Figure 4:**
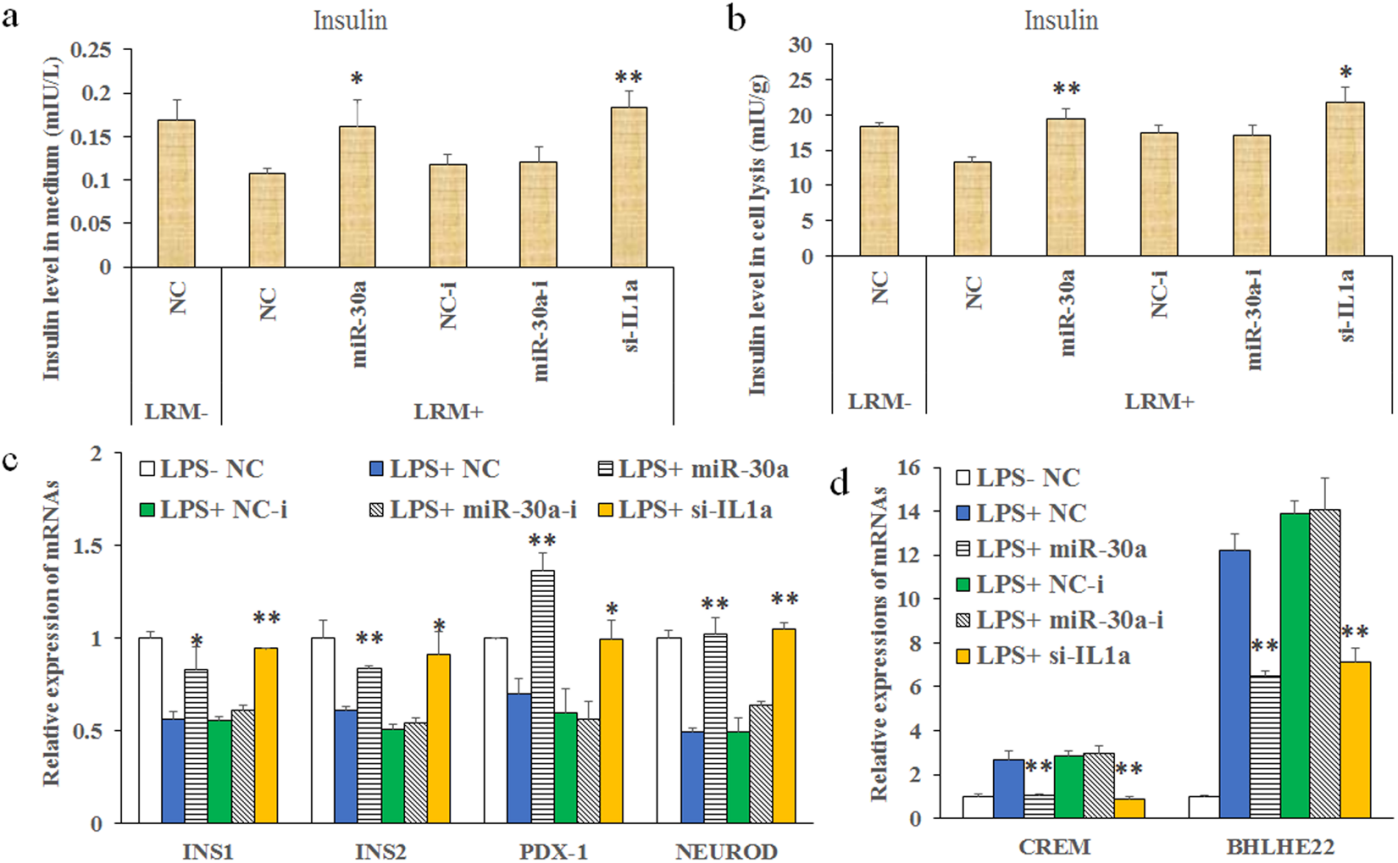
Effects of miR-30a mimics on the insulin levels of cell medium and lysis, as well as mRNA expression in LPS-induced Beta-TC-6 cells. (**a** and **b**) ELISA showing that miR-30a mimics significantly increased the insulin levels of cell medium and lysis in Beta-TC-6 cells after 24 h of LRM incubation; the insulin levels in cell medium indicated the release of intracellular insulin; and the insulin levels in cell lysis indicated the synthesis of intracellular insulin, which was normalized by the cellular protein concentrations. (**c** and **d**) RT-PCR showing that miR-30a mimics significantly upregulated the mRNA expression of Ins1, Ins2, PDX-1 and Neurod, while they dowregulated Crem and Bhlhe22 in Beta-TC-6 cells after 12 h of LRM induction. LRM+/−, incubation with or without LRM; NC, negative controls of miRNA mimics; NC-i, negative controls of miRNA inhibitors or siRNA; miR-30a-i, miRNA-30a inhibitor; and si-IL1a, silencing mRNA fragment of Il-1a. Data are expressed as the Mean ± SD (n = 3); *P < 0.05 and **P < 0.01 vs. NC or NC-i (LPS/LRM+), respectively.

We hypothesized that the anti-inflammatory effects of miR-30a would be resistant to islet dysfunctions induced by inflammatory stimulation because inflammation stimulation increased the oxidative stress and apoptosis in beta-TC-6 cells. We assayed the effects of miR-30a on insulin synthesis and secretion by ELISA. As expected, inflammation stimulation with LRM for 24 h significantly inhibited the intracellular and extracellular insulin levels (Fig. 4a,b). However, miR-30a and si-Il-1a significantly reversed the decreased intracellular and extracellular insulin levels compared with the negative controls. miR-30a inhibitors did not show any significant change.

Furthermore, we assayed the mRNA levels of Insulin 1 and 2 (Ins1 and Ins2), pancreatic and duodenal homeobox 1 (Pdx-1), neurogenic differentiation 1(NeuroD1), cAMP responsive element modulator (Crem), basic helix-loop-helix family, and member e22 (BHLHE22) in beta-TC-6 cells by RT-PCR. Inflammatorystimulation with 12 h of LRM significantly inhibited the expression levels of Ins1, Ins2, Pdx-1, and NeuroD while increasing the expression of Crem and Bhlhe22 (Fig. 4c,d). However, the changes induced by inflammatory stimulation could be effectively reversed by miR-30a and si-Il-1a. miR-30a inhibitors did not show any significant change.

### Effects of miR-30a on the phosphorylated p65 (pp65) and p62 levels in RAW264.7 and Beta-TC-6 cells with inflammatory stimulation

**Figure 5:**
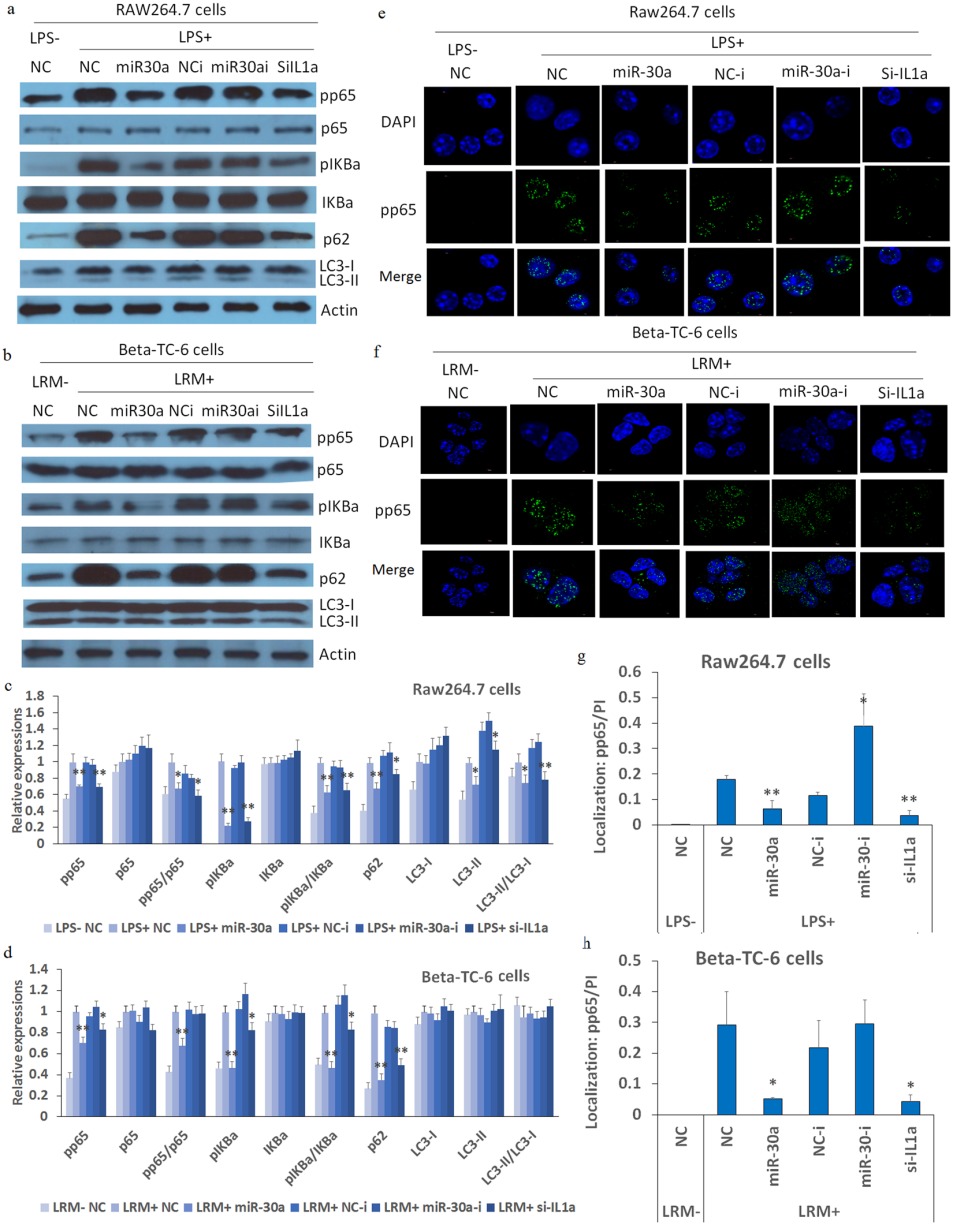
Effects of miR-30a mimics on inflammatory and autophagy factors and NFKB activity in RAW264.7 and Beta-TC-6 cells subjected to inflammatory stimulation. (**a** and **b**) Western blotting showing that miR-30a mimics significantly inhibit the increases of pp65, pIKBa and p62 in RAW264.7 and Beta-TC-6 cells after 24 h of LPS and LRM, respectively (the uncropped images are available in Supplementary Fig. S3); (**c** and **d**) the relative expression was calculated by the ratios of gray density values of protein bands to actin. (**e**–**h**) IF and confocal assays showing that miR-30a mimics significantly inhibited pp65 translocation into the nucleus in RAW264.7 and Beta-TC-6 cells after 24 h of LPS and LRM induction, respectively. The gray density values of fluorescence proteins and DAPI were calculated by ImageJ software, and these protein levels in cells were normalized with DAPI (PI). Approximately 100 cells in each slide (3 slides per group) were randomly selected and the average value in each slide was obtained for further statistical analysis. LRM+/−, incubation with or without LRM; NC, negative controls of miRNA mimics; NC-i, negative controls of miRNA inhibitors or siRNA; miR-30a-i, miRNA-30a inhibitor; and si-IL1a, silencing mRNA fragment of Il-1a. Data are expressed as the Mean ± SD (n = 3); *P < 0.05 and **P < 0.01 vs. NC or NC-i (LPS/LRM+), respectively.

We measured the expression levels of phospho-I Kappa B alpha (pIKBa) and pp65by western blot to assay the potential molecular mechanisms by which miR-30a regulates insulin levels. Considering that autophagy can affect inflammation^22^ and simultaneously regulate insulin function^23^, we also determine two key autophagy factors, p62 and microtubule-associated protein 1A/1B-light chain 3 phosphatidylethanolamine conjugate (LC3-II) levels. In this study, inflammation stimulation with 24 h of LRM significantly increased the pIKBa, pp65, and p62 levels; however, miR-30a and si-Il-1a significantly inhibited these increases (Fig. 5a–d). MiR-30a significantly inhibited the formation of LC3-II in RAW264.7 cells; however, miR-30a did not work in Beta-TC-6 cells. Additionally, miR-30a inhibitors did not show any significant effect. These results indicate that miR-30a regulates NFKB and autophagy pathways in specific cells. However, regulating inflammation instead of autophagy mediated by miR-30a may contribute to its regulation of islet function.

In addition, immunofluorescence (IF) and confocal assay revealed that inflammation stimulation with 24 h of LRM significantly increased the translocation of pp65 from the cytoplasm to the nucleus in RAW264.7 and Beta-TC-6 cells (Fig. 5e–h). Interestingly, miR-30a and si-Il-1a significantly inhibited this translocation. These results further supported that miR-30a inhibits NFKB activity. More specifically, miR-30a inhibitors had a significant effect on pp65 translocation, which required further validation.

### Effects of miR-30a on p62 and insulin localization in Beta-TC-6 cells with inflammatory stimulation

**Figure 6:**
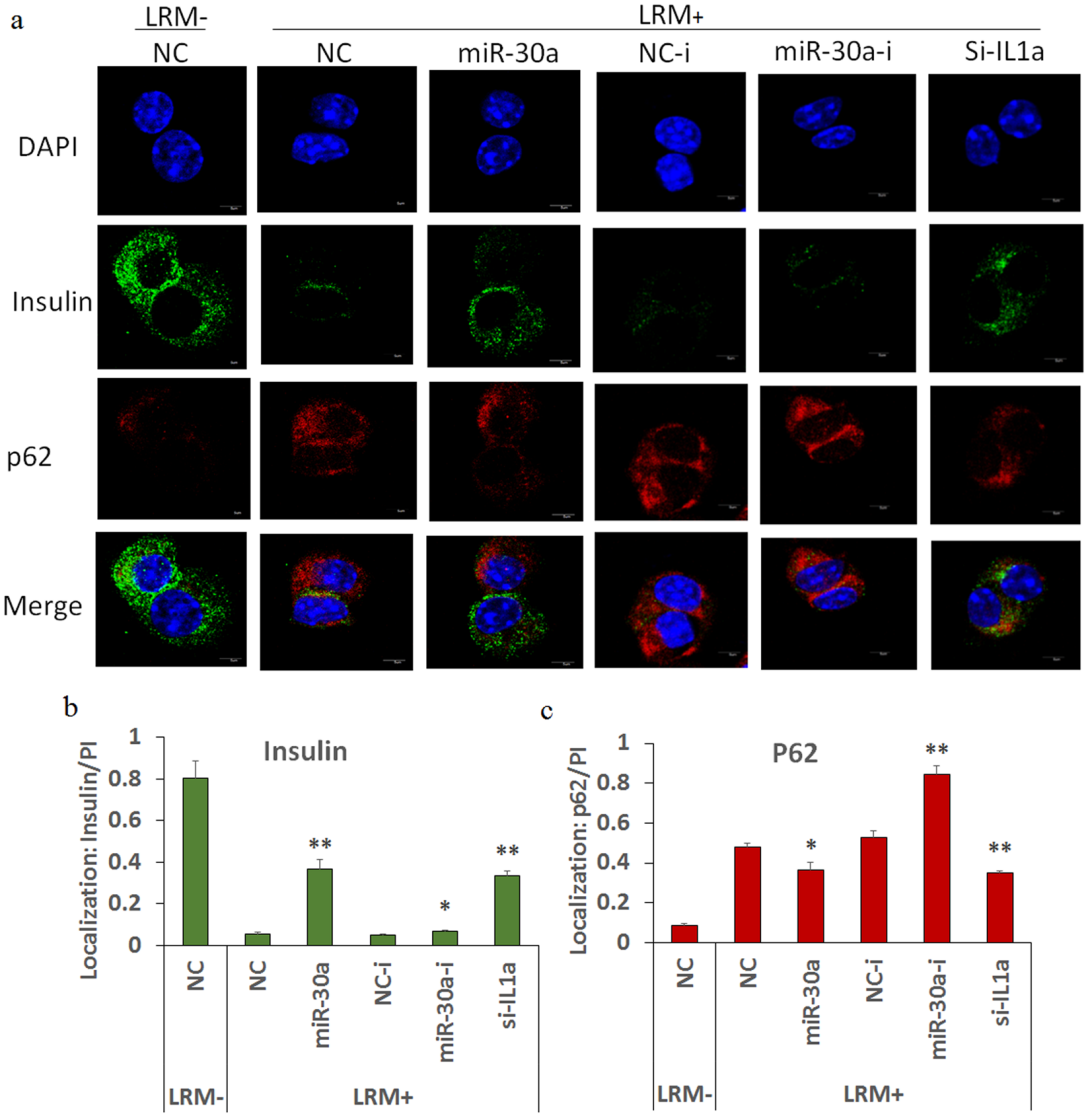
Effects of miR-30a on p62 and insulin localization in Beta-TC-6 cells. (**a**–**c**) IF and confocal assay showing that miR-30a significantly regulates p62 and insulin localization in Beta-TC-6 cells after 24 h of LRM induction; the increased intracellular insulin level with miR-30a might be associated with decreased intracellular p62. The gray density values of proteins and DAPI were calculated by ImageJ software, and these protein levels in cells were normalized with DAPI (PI); approximately 100 cells in each slide (3 slides per group) were randomly selected and the average value in each slide was obtained for further statistical analysis. LRM+/−, incubation with or without LRM; NC, negative controls of miRNA mimics; NC-i, negative controls of miRNA inhibitors or siRNA; miR-30a-i, miRNA-30a inhibitor; and si-IL1a, silencing mRNA fragment of Il-1a. Data are expressed as the Mean ± SD (n = 3); *P < 0.05 and **P < 0.01 vs. NC or NC-i (LPS/LRM+), respectively.

P62 exerts numerous effects, including autophagy and inflammation regulation. In Beta-TC-6 cells, increased p62 was associated with increased formation of p62-positive aggregates, which may mediate insulin degradation as previously reported^23^. Subsequently, we assayed the p62 and insulin levels in Beta-TC-6 cells by IF and confocal assay. The results showed that inflammation stimulation with 24 h of LRM significantly increased p62, while it decreased the insulin levels in Beta-TC-6 cells, whereas miR-30a (also for si-Il-1a) significantly reversed these changes in Beta-TC-6 cells (Fig. 6). Unexpectedly, miR-30a inhibitors significantly increased p62 expression. Meanwhile, p62 demonstrated good co-localization with insulin, indicating that p62-positive aggregates induced by inflammation stimulation recruit insulin and mediate its degradation.

### Endogenous miR-30a expression in RAW264.7 and Beta-TC-6 cells with inflammatory stimulation

**Figure 7:**
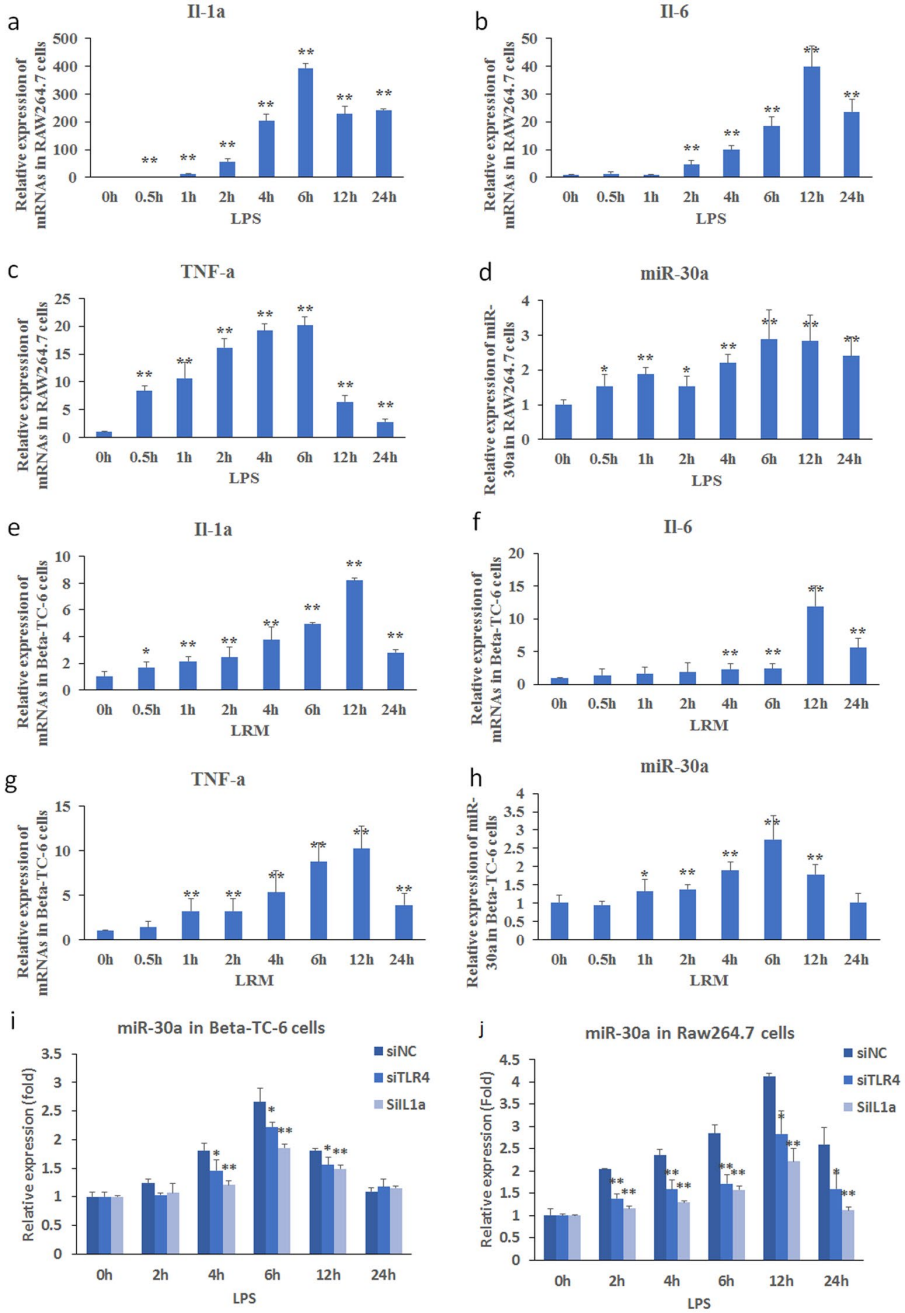
Expressions of inflammatory factors mRNAs and endogenous miR-30a as well as potential signaling pathways in LPS-induced RAW264.7 and LRM-induced Beta-TC-6 cells at different time points assayed by RT-PCR. (**a**–**h**) The expression of Il-1a, Il-6 and TNF-a mRNAs and endogenous miR-30a transiently increased in RAW264.7 cells within 24 h of LPS and Beta-TC-6 cells within 24 h of LRM stimulation, respectively. (**i** and **j**) miR-30a expression depends on the TLR4 and IL-1α pathways in RAW264.7 cells within 24 h of LPS (**i**) and Beta-TC-6 cells within 24 h of LRM stimulation (**j**). LPS+/−, incubation with or without LPS; LRM+/−, incubation with or without LRM; siNC, negative controls of siRNA; siTLR4, silencing mRNA fragment of Tlr-4; and si-IL1a, silencing mRNA fragment of Il-1a. Data are expressed as Mean ± SD (n = 3); *P < 0.05, **P < 0.01 the mice after LPS induction (at different time points) vs. controls before LPS induction (0 h, see 7a-h) or *P < 0.05 and **P < 0.01 siRNAs vs. negative controls (see 7i and 7j).

We assayed the endogenous miR-30a expression in RAW264.7 and Beta-TC-6 cells with inflammatory stimulation. RT-PCR results showed that inflammation stimulation significantly upregulated the expression of miR-30a and inflammatory factors (Il-1a, Il-6 and TNF-a) in RAW264.7 (Fig. 7a–d) and Beta-TC-6 cells (Fig. 7e–h). MiR-30a levels were concomitantly changed with Il-1a, Il-6, and TNF-a levels.

Inflammation significantly increased endogenous miR-30a expression. Subsequently, knockdown of TLR4 and IL-1α using si-TLR4 and si-Il-1a significantly inhibited the increase in endogenous miR-30a in RAW264.7 and Beta-TC-6 cells (Fig. 7i–j). These results indicate that endogenous miR-30a depends on TLR4- and IL-1α-mediated inflammatory pathways.

### Endogenous miR-30a expression in PBMCs and pancreatic tissues of LPS- and streptozotocin (STZ)-induced mice

**Figure 8:**
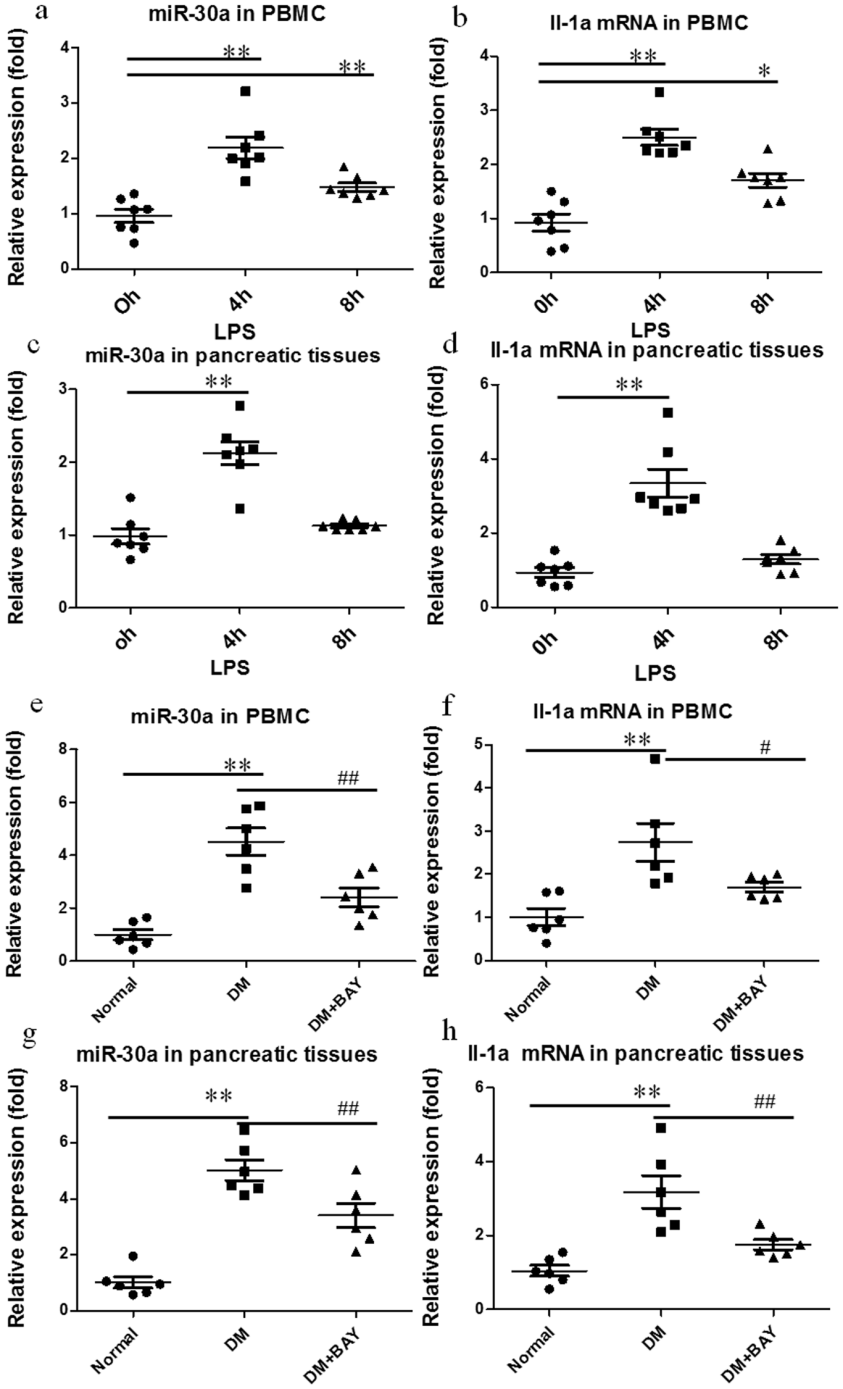
Endogenous miR-30a and Il-1a mRNA expression levels in PBMC and pancreatic tissues in LPS- and STZ-induced mice assayed by RT-PCR. (**a**–**d**) Endogenous miR-30a and Il-1a mRNA expression levels showing a transient increase in PBMCs and pancreatic tissues from LPS (2 mg/kg, ip)-induced mice within 8 h; (**e**,**f**) endogenous miR-30a and Il-1a mRNA expression levels showing a significant increase in PBMCs and pancreatic tissues of STZ (100 mg/kg, ip)-induced mice after 4 weeks, but this increase was significantly attenuated by BAY117082 (5 mg/kg, ip), an NFKB inhibitor. Normal, normal control mice; DM, diabetic control mice; and DM+BAY, diabetic mice treated by BAY117082. Data are expressed as the Mean ± SD (n = 6–7); for LPS induced mice, **P < 0.01 for mice after LPS treatment (4 h or 8 h) vs. controls before LPS treatment (0 h) (see 8a–d). For STZ-induced diabetic mice, **P < 0.01 for diabetic control mice vs. normal control mice, and ^#^ *P* < 0.05 and ^##^ *P* < 0.01 for diabetic mice treated by BAY117802 vs. diabetic control mice (see 8e–h).

To validate whether miR-30a was common in related tissues subjected to inflammatory stimulation *in vivo*, we assayed the expression levels of endogenous miR-30a and Il-1a mRNA in PBMCs and pancreatic tissues of LPS- and STZ-induced mice by RT-PCR. After the intraperitoneal injection of 2 mg/kg LPS, the mice showed a significant transient increase in miR-30a and Il-1a in PBMCs and pancreatic tissues at 4 h compared to 0 h (Fig. 8a–d). After 4 weeks of intraperitoneal (IP) injection of 100 mg/kg STZ, miR-30a and Il-1a significantly increased in PBMCs and pancreatic tissues of mice (Fig. 8e–h); however, this increase was effectively inhibited by BAY117082 (5 mg/kg, IP), an NFKB inhibitor^24^. Both LPS and STZ can induce systemic inflammation in animals *in vivo*. These results further support that inflammation can promote endogenous miR-30a expression even in tissues mixed with multiple cell sources and this increase in miR-30a might play a key role in inflammatory regulation in all kinds of tissues *in vivo* and serve as an important inflammatory biomarker. However, we need to further validate these results by using other or pure cell populations in animals or humans.

## Discussion

In this study, IL-1α knockdown by miR-30a and si-Il-1a exhibited a significant attenuation of NFKB activation, oxidative stress, and inflammation-induced apoptosis and death. However, miR-30a inhibitors, except pp65 and p62, cannot exert significant effects in most experiments. Possible reasons for this were 1) the compensatory effects of endogenous miR-30a were still too weak to attenuate strong inflammatory stimulation and 2) the abundance of endogenous miR-30a was sufficiently low that its inhibition was not effective. Despite this, exogenous miR-30a mimics can exert significant effects in most cases. Increased oxidative stress, apoptosis, and death may reduce the production and secretion of insulin in beta islet cells^25^. Therefore, the effects of miR-30a on insulin levels may be associated with its anti-inflammatory activities.

Transcription factor p65 is a subunit of NFKB factors encoded by the RELA gene^26^. pIKBa cannot inhibit p65, but it leads to p65 phosphorylation and translocation from the cytoplasm to the nucleus, which may activate the expression of inflammatory factors^27^. NFKB activation not only impairs insulin sensitivity^28^, it induces beta cell death or apoptosis^29^, which might cause the low expression of insulin-related factors. Ins1, Ins2, PDX-1, and Neurog3 are the key factors responsible for insulin production and beta-islet function^30^. CREM and BHLHE22 are the negative regulators of insulin factors^31^. In the present study, inflammation inhibited the expression levels of Ins1, Ins2, Pdx-1, and Neurog3, and it promoted those of Crem and Bhlhe22, which may account for the low insulin levels caused by inflammation in beta islet cells. Interestingly, miR-30a can reverse these changes caused by activation of the NFKB pathway and inflammation stimulation.

Insulin is produced by, and degraded within, pancreatic beta cells^32^. In the present study, we investigated whether insulin degradation is also regulated by miR-30a. The ubiquitin–proteasome system and autophagy are the two main proteolytic systems^33^. P62 not only contributes to autophagy, it mediates protein degradation. The inflammation activated by the TLR4 pathway significantly increases p62 transcription and translation^34^ and might mediate autophagic degradation. Furthermore, increased p62 with inflammatory stimulation may increase insulin degradation because autophagy can regulate beta cell insulin homeostasis, as previously described^23^, which may explain why inflammation causes low insulin levels in islet cells. Although the LC3-II levels did not significantly change in Beta-TC-6 cells, p62 significantly increased and may trigger another proteolytic system involved in insulin degradation. Reduced p62 by miR-30a might inhibit the degradation of insulin and increase insulin levels. However, the exact mechanisms should be further investigated.

Inflammation usually triggers an anti-inflammatory reaction to maintain homeostasis and avoid overreaction and damage^35^. Endogenously produced lipid mediators (lipoxins, resolvins, protectins, and maresins)^36^, proteins (annexin A1 and galectins), peptides, gaseous mediators (hydrogen sulfide), purine (adenosine), and neuromodulators^37^ contribute to the resolution of inflammation. For the IL-1α family, there is a balance between pro-inflammatory and anti-inflammatory responses^38^. IL-1 cytokines induce potent inflammatory responses, but they are tightly controlled by naturally occurring inhibitors^39^. However, only a few studies have reported miRNA-mediated inflammatory resolution, although miRNAs serve as powerful regulators in the pathogenesis of inflammatory diseases^40^. In the present study, we found that endogenous miR-30a increased with increasing inflammatory factors. The increased expression of miR-30a may be a homeostasis response to inflammatory stimulation because miR-30a can exert anti-inflammatory effects. Although this effect of miR-30a may be ignored in resisting strong inflammation stimulation, constellation effects of many similar tiny activities of miRNAs may contribute to detectable effects. In our previous studies, we found a similar homeostasis regulation for miR-181a and let-7^41, 42^. In the present study, we speculate that miR-30a is an important inflammation buffer factor; however, it needs to be further investigated.

In particular, miR-30a increased with increasing IL-1α in 1) LPS-induced RAW264.7 and LRM-induced Beta-TC-6 cells *in vitro* and, 2) PBMCs and islets in LPS- or STZ-induced mice. Therefore, miR-30a may be developed as an important biomarker for inflammation stress. Diabetes is a low-grade chronic inflammatory disease, while lung infection is an acute inflammatory disease. The TLR4 pathway is activated in diabetes^43^. For lung-infection patients, LPS released by bacteria might activate the TLR4 pathway and mediate lung injuries^44^. Therefore, miR-30a might be an important inflammation response factor mediated by the TLR4 pathway.

The activation of the TLR4 pathway can enhance NFKB activity and promote Il-1a transcription^45^. Produced pre-IL-1α can be processed into mature IL-1α and then secreted and act on the extracellular IL-1α receptor. Sequentially, produced pre-IL-1α can enhance NFKB activity and then contribute a vicious cycle of inflammatory signaling pathways. Theoretically, the inhibition of TLR4 or IL-1α would attenuate the inflammation response. In the present study, the knockdown of TLR4 and IL-1α significantly inhibited miR-30a expression, suggesting that the TLR4 and IL-1α pathways regulate miR-30a expression.

LPS significantly activates NFKB activity^46^ and then promotes the transcription of Il-1a by activating TLR4. Increased or decreased Il-1a mRNA expression by inflammatory stimulation might indicate a potential increase or decrease in NFKB activity. In this study, we observed a transient increase in the Il-1a mRNA level, which was accompanied by a transient increase of miR-30a within 8 h of LPS treatment and indirectly suggests that NFKB activity might invovle miR-30a expression. STZ-induced diabetic animals also had increased NFKB activity as previously reported^47^. BAY117082 is a strong inhibitor of NFKB activity^24^. Interestingly, in this study, we used the NFKB inhibitor in STZ-induced mice and found that miR-30a expression was significantly controlled by NFKB activity. Taken together, we suspected that endogenous miR-30a expression was regulated by the TLR4/IL-1α/NFKB pathways. However, the exact molecular mechanisms should be further investigated.

In conclusion, the main findings of this manuscript include the following (Supplementary Fig. S4).

1. MiR-30a can inhibit the expression of IL-1α by binding to the Il-1a-3′-UTR. Then, miR-30a can inhibit the development of inflammation-induced oxidative stress and apoptosis in immune cells and in beta islet cells. These results suggest that miR-30a systemically controls the inflammation micro-environment and protects islet cells. Therefore, miR-30a might serve as an important anti-inflammatory factor that attenuates the influence of inflammation on diabetes development.
2. The protective effects of miR-30a on islets were associated with a decrease in IL-1α, pp65, and p62 and an increase in insulin. In other words, the “miR-30a/IL-1α/p65/p62/insulin” axis may be an important pathway that regulates islet inflammation and functions, and it could be used as a novel antidiabetic tactic.
3. MiR-30a can be induced by inflammatory stimulation to serve as an inflammation-resolving buffer factor and exhibit a homeostasis response that depends TLR4/IL-1α/NFKB pathways. Based on translational medicine, miR-30a may serve as a promising biomarker and a response factor for acute and chronic inflammatory diseases. However, the exact molecular mechanisms of miR-30a–inflammation interactions and potential pharmacological and clinical applications of miR-30a in acute and chronic inflammatory diseases (bacterial infection and diabetes) should be further investigated in the future.

## Methods

### Cell culture and inflammation induction

RAW264.7 and Beta-TC-6 cells were provided by the Cell Resource Center of the Shanghai Institute for Biological Sciences, Chinese Academy of Sciences, China. Both cells types were cultured in Dulbecco’s Modified Eagle’s Medium (DMEM, high glucose, Gibco®, ThermoFisher Scientific, USA) supplemented with 10% fetal bovine serum (FBS, Premium, Pan Biotech) and 1% Pen-strep antibiotics (Gibco™, ThermoFisher Scientific, USA); then, they were incubated in a humidified atmosphere of 5% CO_2_ at 37 °C. The cells were seeded into six-well plates at a density of 2.5 × 10^5^ cells per well.

For RAW264.7 cells, inflammation was induced by adding 2 µg/mL LPS (Lot. No. L4391, Sigma–Aldrich, USA) after 12 h of attachment. For Beta-TC-6 cells, inflammation was induced by a mixture of LRM and common medium at 1:3 ratio (v:v). LRM was prepared by adding 2 µg/mL LPS to RAW264.7 cell medium and then collecting the supernatant medium after incubation with LPS for 12 h.

For mRNA extraction, cell samples were collected at 0, 0.5, 1, 2, 4, 6, 12, and 24 h after LPS induction. In brief, cells were washed twice with ice-cold PBS, and 1 mL of RNA isolation reagent (TRIzol™, Invitrogen™, ThermoFisher Scientific, USA) was added to the wells for cell RNA extraction.

For protein collection, after the medium samples were collected (if necessary), cells were washed with ice-cold PBS twice. A 200-µL aliquot of cell lysis buffer (50 mM Tris–HCl, 4 M urea, and 1% Triton X-100, pH 8.0) was then added to the wells for cell sample collections. All samples were immediately stored at −80 °C for future biochemical assays.

### Luciferase reporter assays

Luciferase assays were performed as previously described^48^. In brief, the UTRs of murine Il-1a contain the miR-30a binding site, and their corresponding mutated UTRs were amplified by PCR using the primers shown in supplementary Table S2. These target sequences were cloned into the pRL-TK reporter vectors (Promega, USA). 293T cells were seeded into 24-well plates at a density of 5 × 10^4^. Co-transfection was performed the following day using 300 ng of constructed plasmid and 20 pmol of each miRNA with Lipofectamine 2000. Cell lysates were collected after 24 h of transfection. Renilla luciferase activities were measured using a Luciferase Reporter Assay System (Promega, USA). Each experiment was conducted in triplicate on a Thermo Scientific Varioskan Flash spectral scanning multimode reader (USA). The total protein concentration was determined at 595 nm by using the Bradford assay (Bio-Rad) on a spectrophotometer (TECAN, Switzerland). Luciferase activity was normalized by the total protein content.

The cloned sequence:

TATTTCGGGAGTCTATTCACTTGGGAAGTGCTGACAGTCTGTATGTACCATGTACAGGAACCTTCCTCACCCTGAGTCACTTGCACAGCATGTGCTGAGTCTCTGTAATTCTAAATGAATGTTTACCCTCTTTGTAAGAGAAGAGCAAACCCTAGTGGAGCCACCCCGACATATGATACTATCTGTTATTTTAAAGAGTACCCTATAGTTTGCTCAGTACTAATCATTTTAATTACTATTCTGCATGGCTTCTTAGGAGGATCAAAAAGACTCTA.

The mutated sequence:

TATTTCGGGAGTCTATTCACTTGGGAAGTGCTGACAGTCTGTATGTACCATGTACAGGAACCTTCCTCACCCTGAGTCACTTGCACAGCATGTGCTGAGTCTCTGTAATTCTAAATGAAAGCTAAGCCTCTTTGTAAGAGAAGAGCAAACCCTAGTGGAGCCACCCCGACATATGATACTATCTGTTATTTTAAAGAGTACCCTATAGTTTGCTCAGTACTAATCATTTTAATTACTATTCTGCATGGCTTCTTAGGAGGATCAAAAAGACTCTA.

### miRNA or siRNA transfection

miRNA mimics or siRNA duplexes were synthesized by Shanghai GenePharma Co. (Shanghai, China) (Supplementary Table S1). Il-1a siRNA [(m) sc-39614] was purchased from Santa Cruz Biotechnology, Inc., USA. miRNA mimics or siRNA duplexes with random sequence were used as negative controls. RAW264.7 or Beta-TC-6 cells at a density of 2.5–5 × 10^5^ were seeded into six-well plates for 12 h of attachment. The cells were transferred onto a fresh medium (DMEM + 10% FBS) and then transfected with siRNA or miRNA mimics at a concentration of 50 pmol/well to 60 pmol/well using Lipofectamine 2000 (Invitrogen^TM^, USA) in accordance with the manufacturer’s instructions. After 6–12 h of transfection, inflammation was induced by exchanging fresh medium containing 2 µg/mL LPS in RAW264.7 cells or a mixture of LRM and fresh medium (1:3 v/v) in Beta-TC-6 cells. The cell medium and cell pellet samples were then collected in accordance with the aforementioned protocol after 24 h of inflammatory induction.

### miRNA and mRNA q-PCR

miRNA and mRNA q-PCR were conducted as previously described^48^. The primers for the mRNA assays were synthesized from Invitrogen (Supplemental Table S3). More details are available in Supplementary method S2.

### ELISA

Murine IL-1α, IL-6, and TNF-α were assayed using the QuickEIATM ELISA method (DAKEWE Biotech Co., Ltd., Beijing, China). Murine insulin was assayed using an ELISA kit (Shanghai Westang Bio-tech, China). Total protein concentration was determined at 595 nm using the Bradford assay (Bio-Rad, USA) on a spectrophotometer (TECAN, Switzerland). ELISA data of cell lysate samples were normalized with protein concentrations. Other steps were performed as described in the protocols accompanying the kits.

### ROS assay

ROS were assayed using a kit provided by the Beyotime Institute of Biotechnology (Haimen, Jiangsu, China) as previously described^48^. More details are available in Supplementary method S1.

### Flow cytometry assay

After transfecting with miRNA mimics and siRNA, RAW264.7 and Beta-TC-6 cells were treated with LPS or LRM for 24 h as described above. Then, the cells were collected and dual stained with Annexin V and propidium iodide (PI, V13241, Dead Cell apoptosis kit with Annexin V Alexa Fluor™ 488 & PI, InvitrogenTM, ThermoFisher Scientific, USA) for 30 min at room temperature. The stained cells were immediately analyzed by flow cytometry (Becton Dickinson, USA).

### Western blot analysis

The details of the western blot analysis are available in Supplementary method S3.

### IF and confocal assay

An IF assay in RAW264.7 and Beta-TC-6 cells was performed as previously described^49^. First, the cells were transfected with miRNA mimics or siRNA as described above. Subsequently, circular transparent glass slides (diameter of 10 mm) were placed at the bottom of a six-well plate. Cells at a density of 2.5 × 10^5^/well (2 mL of cell culture medium) were added to the surface of glass slides in a six-well plate. The cells were cultured in fresh medium (DMEM + 10% FBS). After 24 h, the immobilized cells in the slides were washed with PBS and then fixed with 4% paraformaldehyde in PBS. The cells were washed with PBS three times and then incubated with 0.1% Triton in PBS for 15 min. After three washes with PBS, the cells were blocked with 3% bovine serum albumin in PBS for 1 h. The cells were incubated with goat polyclonal antibody against phospho-p65 (1:100, #3039, Cell Signaling Technology, USA), insulin (1:100, Sc-9168, Santa Cruz Biotechnology, INC., USA), or mouse polyclonal antibody against p62 (1:100, Cat No. 610832, BD Biosciences, USA) in PBS for 1 h and then washed three times with PBS. The cells were incubated with goat anti-rabbit IgG H&L (1:100, ab150077, Alexa Fluor® 488, Abcam, UK) or goat anti-mouse (1:100, ab150119, Alexa Fluor® 647, Abcam, UK) in PBS for 1 h. The fluorescence signals of the cells were imaged by confocal microscopy (OLYMPUS, Japan) and assayed by FV10-ASW Viewer 3.1 and ImageJ software.

### LPS- and STZ-induced mice

Four-week-old male NIH mice were obtained from the Guangdong Medical Animal Center (Guang Zhou, China). Animals were kept in an environmentally controlled breeding room (temperature: 20 ± 2 °C; humidity: 60% ± 5%; 12 h dark/light cycle). The animals were fed standard laboratory chow diets with water ad libitum and fasted from 9:00 am to 3:00 pm before the experiments. The study was performed in strict accordance with the recommendations of the Guide for the Care and Use of Laboratory Animals of the Institutional Animal Care and Use Committee of Tsinghua University. The protocol was approved by the Animal Welfare and Ethics Committee of Tsinghua University, China. All surgeries were performed under sodium pentobarbital anesthesia, and efforts were made to minimize suffering. After housing for one week, the LPS-induced mice were IP injected with 2 mg/kg LPS (Sigma–Aldrich, USA) for acute inflammation stimulation. Blood was collected once from the orbital plexus at 0, 4, and 8 h. PBMCs were immediately separated by using the appropriate kit (Tian Jin Hao Yang Biological Manufacture Co., Ltd.) and then used for miRNA and protein extraction. The mice were sacrificed, and pancreatic tissues were removed and frozen in liquid nitrogen. The samples were stored at 80 °C until the biochemical assays. The STZ-induced mice were IP injected with 100 mg/kg STZ (Sangon Biotech, Shanghai, China) as previously described^50^. After a week of STZ administration, mice with a blood glucose level of more than 11.1 mmol/L were used as diabetic models. After 2 weeks, the STZ-induced mice were IP injected with a NFKB inhibitor, BAY117082 (Cat No. 1956772, PeproTech-BIOGEMS, USA) for a continuous week (5 mg/kg, twice a week). Stocking solution of BAY117082 was firstly dissolved in DMSO and then diluted with PBS 1000-fold before use. Normal and diabetic controls were treated with DMSO-PBS (0.1%) with identical volume.Then, the mice were sacrificed to collect blood and pancreatic tissues as described above.

### Statistical Analysis

Data are expressed as the mean ± SD. Statistical significance of data was evaluated by one-way ANOVA. Newman–Keuls comparison was used to determine the source of significant difference where appropriate. Statistical significance was considered for *P* < 0.05.

## Electronic supplementary material

Supplementary information
