# Supplementary material for: MiR-30a targets IL-1α and regulates islet functions as an inflammation buffer and response factor
Source: Sci Rep. 2017 Jul 13;7:5270. doi: 10.1038/s41598-017-05560-1 (PMC5509704; doi:10.1038/s41598-017-05560-1)
Supplement: Supplementary file 1 — Supplementary information [file 41598_2017_5560_MOESM1_ESM.pdf]

MiR-30a targets IL1a and regulates islet functions as an inflammation buffer and response factor

Xin Jiang, Chenke Xu, Fan Lei, Liao Meijian, Wei Wang, Naihan Xu, Yaou Zhang, Weidong Xie

Supplementary Fig S1

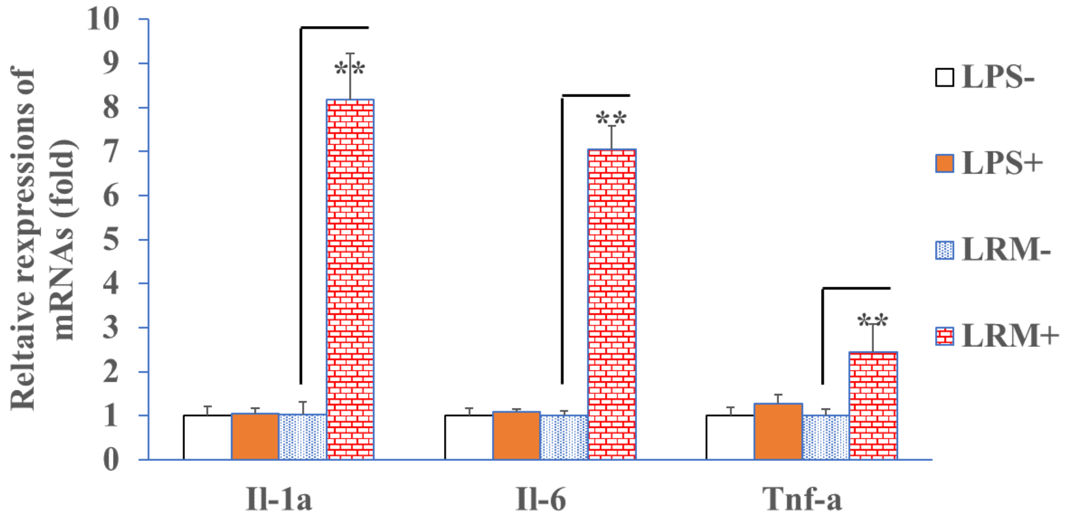

Supplementary Fig. S1. mRNA expressions of inflammatory factors in LPS- and LRM-induced Beta-TC-6 cells. LRM-treated Beta-TC-6 cells showing a significant increase in Il-1a, Il-6 and TNF-a mRNAs expression levels, while LPS does not have a significant effect. LPS+/−, incubation with or without 2 μg/ml LPS for 12 h; LRM+/−, incubation with or without LRM (2 μg/ml LPS induced RAW264.7 cell culturing medium mixed with common cell medium at a ratio of 1:3(v/v)) for 12 h. Data are expressed as Mean±SD (n=3); *P <0.05 and **P < 0.01 vs LRM−.

MiR-30a targets IL1a and regulates islet functions as an inflammation buffer and response factor

Xin Jiang, Chenke Xu, Fan Lei, Liao Meijian, Wei Wang, Naihan Xu, Yaou Zhang, Weidong Xie

Supplementary Fig S2

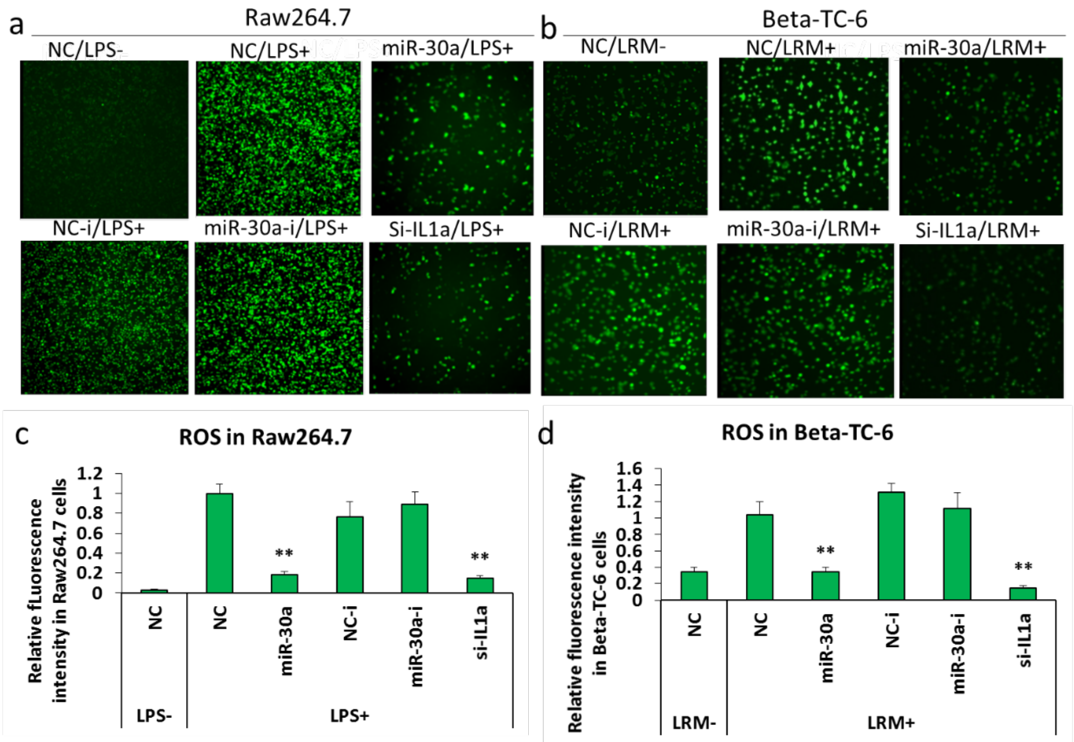

Supplementary Fig. S2. Effects of miR-30a mimics on ROS in LPS-induced RAW 264.7 and Beta-TC-6 cells. (a and b) Fluorescence microscopy showing that miR-30a significantly inhibited the production of ROS in both LPS-induced RAW264.7 cells and LRM-induced Beta-TC-6 cells. (c and d) Relative fluorescence intensity assay showing that miR-30a significantly inhibited the production of ROS in both LPS-induced Raw264.7

cells and LRM-induced Beta-TC-6 cells. LPS+/−, incubation with or without LPS; LRM+/−, incubation with or without LRM; NC, negative controls of miRNA mimics; NC-i, negative controls of miRNA inhibitors or siRNA; miR-30a-i, miRNA-30a inhibitor; and si-IL1a, silencing mRNA fragment of Il-1a. Data are expressed as the Mean ± SD (n=3); ** $P < 0.01$ vs. NC or NC-i (LPS/LRM+), respectively.

MiR-30a targets IL1a and regulates islet functions as an inflammation buffer and response factor

Xin Jiang, Chenke Xu, Fan Lei, Liao Meijian, Wei Wang, Naihan Xu, Yaou Zhang, Weidong Xie

Supplementary Fig S3

Supplementary Fig S3 (a)

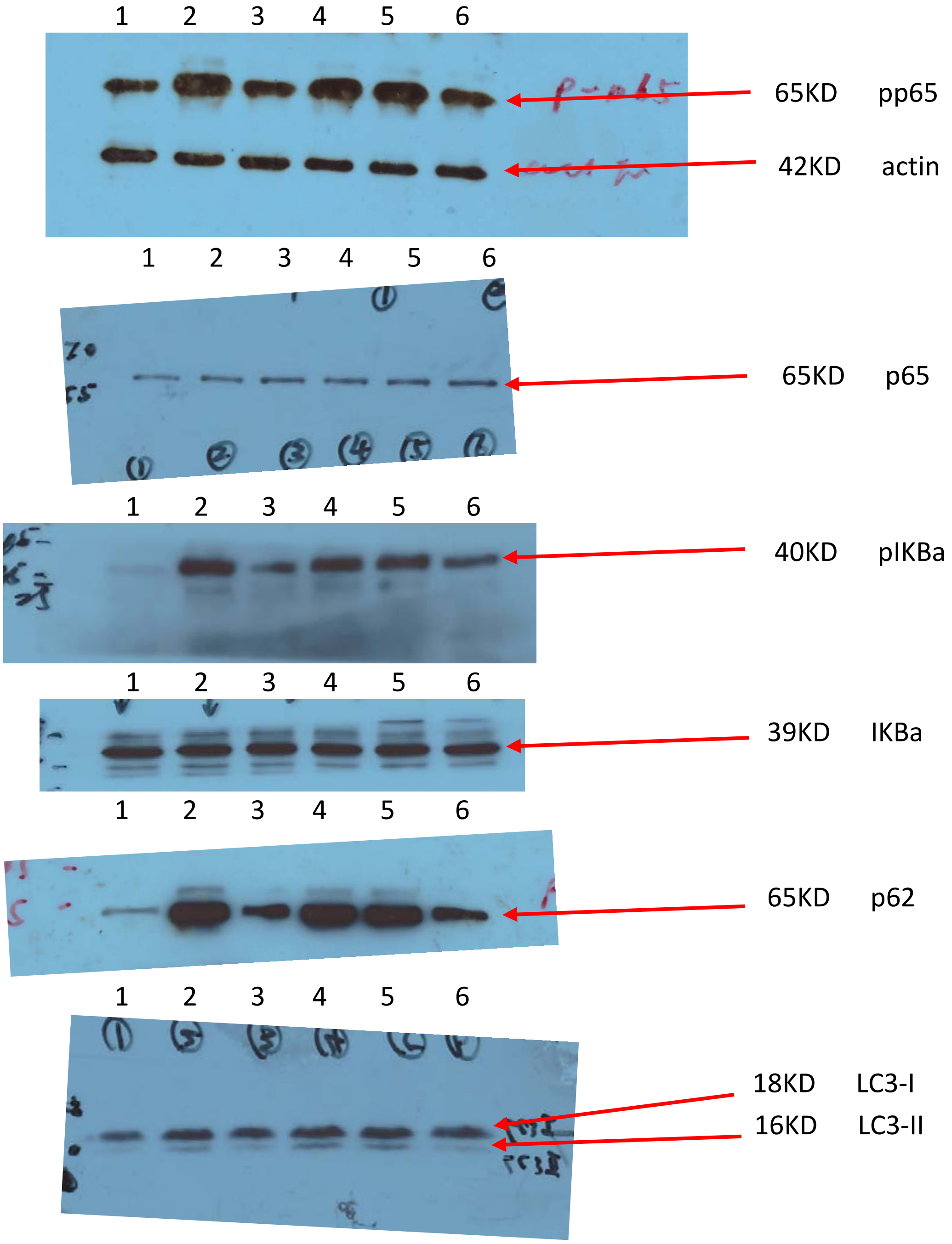

Supplementary Fig S3 (a) The uncropped images of Western blotting in Raw24.7 cells. Western blotting showing that miR-30a mimics significantly inhibit the increases of pp65, pIKBa and p62 in RAW264.7 and Beta-TC-6 cells after 24 h of LPS and LRM, respectively. 1 LPS-NC, 2 LPS+NC, 3 LPS+miR30a, 4 LPS+NCi, 5 LPS+miR30ai, 6 LPS+siIL1a

Supplementary Fig S3 (b)

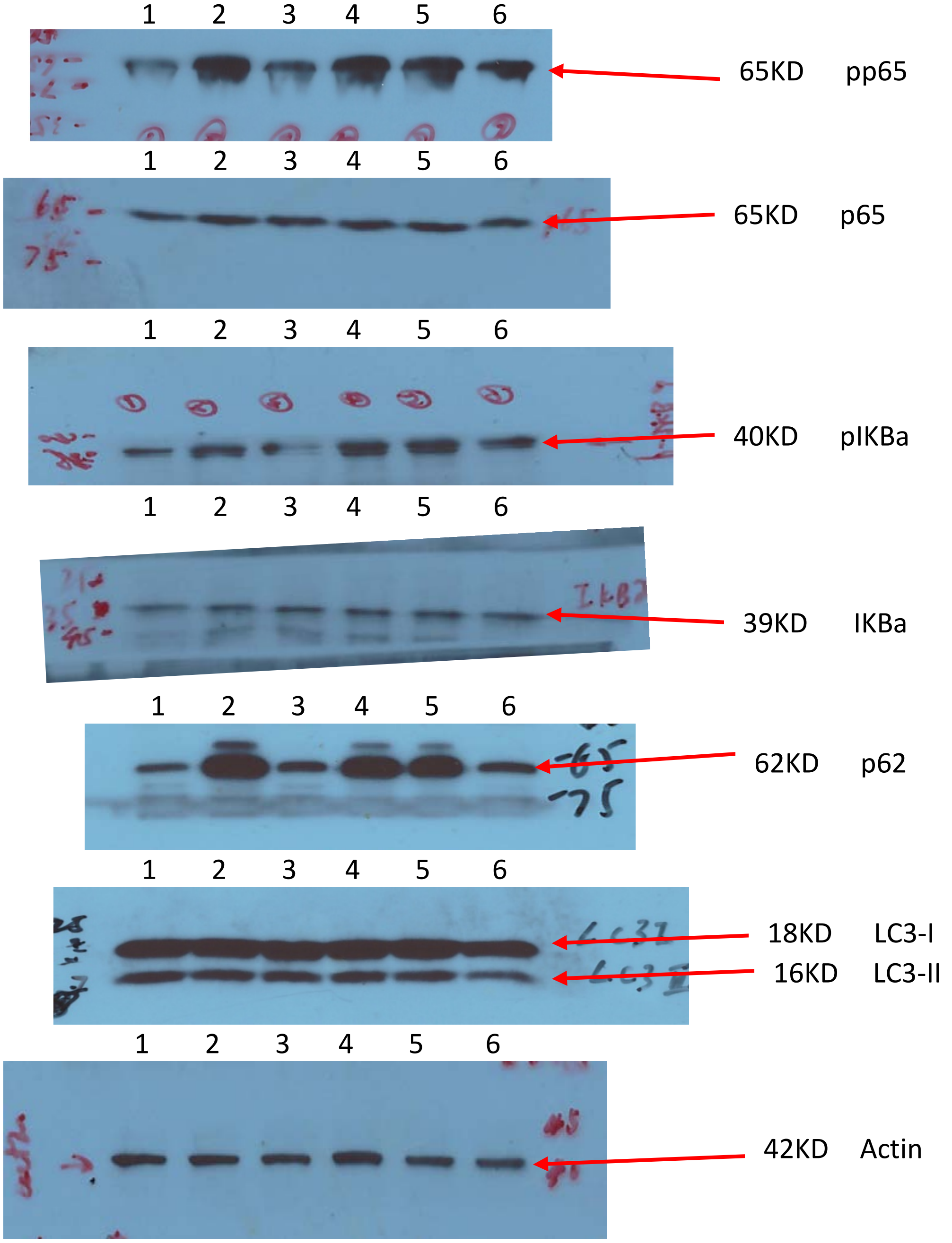

Supplementary Fig S3 (b) The uncropped images of Western blotting in Beta-TC-6 cells. Western blotting showing that miR-30a mimics significantly inhibit the increases of pp65, pIKBa and p62 in Beta-TC-6 cells after 24 h of LRM. 1 LRM-NC, 2 LRM+NC, 3 LRM+miR30a, 4 LRM+NCi, 5 LRM+miR30ai, 6 LRM+siIL1a

MiR-30a targets IL1a and regulates islet functions as an inflammation buffer and response factor

Xin Jiang, Chenke Xu, Fan Lei, Liao Meijian, Wei Wang, Naihan Xu, Yaou Zhang, Weidong Xie

Supplementary Fig S3

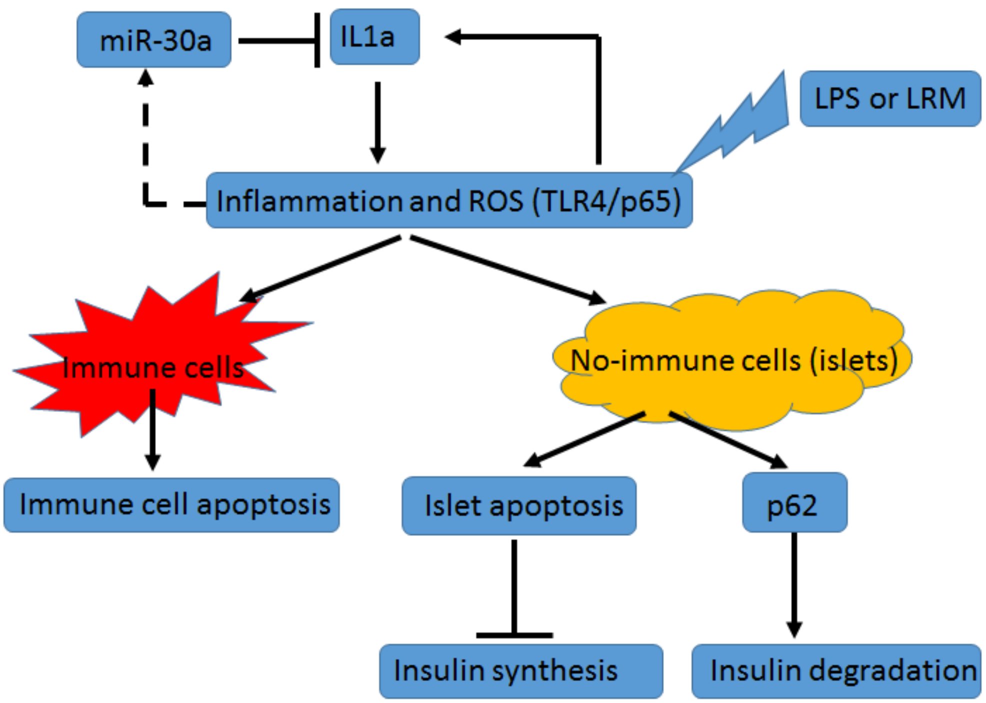

Supplemental Fig S3. Summary of effects and potential mechanisms of miR-30a on inflammation regulation and islet protection.

MiR-30a targets IL1a and regulates islet functions as an inflammation buffer and response factor

Xin Jiang, Chenke Xu, Fan Lei, Liao Meijian, Wei Wang, Naihan Xu, Yaou Zhang, Weidong Xie

Supplementary Method S1

ROS assay

In brief, the 2,7-Dichlorodi-hydrofluorescein diacetate (DCFH-DA) fluorescence probe was transported into the cells and then hydrolyzed into 2,7-dichlorodi-hydrofluorescein (DCFH). Intracellular active oxygen binds to DCFH and causes DCFH to emit fluorescence, which can be detected through fluorescence microscopy (excitation wavelength: 485 nm; emission wavelength: 525 nm). In this study, RAW264.7 and Beta-TC-6 cells (5×10^4 per well) were seeded into 24-well plates for 12 h of attachment. After replacing the medium, the cells were transfected with miRNA or siRNA mimics for 12 h. Subsequently, LPS (2 mg/mL final concentration) and LRM (1:3, v/v) was added to the medium with RAW264.7 and Beta-TC-6 cells for 6–24 h after medium replacement, respectively. Subsequently, the medium from each plate was replaced with fresh medium (without FBS) containing DCFH-DA (10 mM final concentration), and the plates were incubated for 20 min. The cells were then washed three times with fresh medium (without FBS) and immediately observed under a fluorescence microscope magnified at 200× (LEICA DMI6000B, German). Six random areas with the same size from the captured image of each sample were selected. The gray density of the fluorescence intensity in each area was calculated using ImageJ software, and the average value of each sample was obtained with three biological

repetitions for further statistical analysis. We defined the average gray density values of fluorescence intensity from negative controls of miRNAs treated with LPS or LRM as 1, and all values were normalized to this value.

MiR-30a targets IL1a and regulates islet functions as an inflammation buffer and response factor

Xin Jiang, Chenke Xu, Fan Lei, Liao Meijian, Wei Wang, Naihan Xu, Yaou Zhang, Weidong Xie

Supplementary Method S2

miRNA and mRNA q-PCR

For the miRNA assay, miR-30a was analyzed using the miRNA assay kit (GenePharma, Shanghai, China) in accordance with the manufacturer's instructions. The U6 gene was used as an internal control for normalization. In brief, the reverse transcription (RT) reaction was performed using a PrimeScript™ First Strand cDNA Synthesis Kit (Takara, Dalian, China). PCR was confirmed by SYBR Green I dye (Takara, Dalian, China) with an ABI PRISM 7300 Real-time PCR System (Applied Biosystems, USA). The primers for the mRNA assay were synthesized from Invitrogen (supplemental Table S2). Actin was used as an internal control for normalization. RT was performed using a PrimeScriptTM 1st Strand cDNA Synthesis Kit (Takara, Dalian, China) in accordance with the manufacturer's instructions. Q-PCR analysis was conducted using SYBR® Green I dye according to the manufacturer's protocol (Takara, Dalian, China) in an ABI PRISM 7300 Real-time PCR System (Applied Biosystems, USA). The fold change was calculated using the 2^{-ddCt} method of relative quantification. All experiments were conducted in triplicate.

MiR-30a targets IL1a and regulates islet functions as an inflammation buffer and response factor

Xin Jiang, Chenke Xu, Fan Lei, Liao Meijian, Wei Wang, Naihan Xu, Yaou Zhang, Weidong Xie

Supplementary Method S3

Western blot analysis

Cell lysis extracts were separated by SDS-PAGE (12%) and transferred onto nitrocellulose transfer membranes (PALL 66485, BioTrace NT, USA). The membranes were blocked with 5% non-fat dry milk for 1 h and left to react overnight at 4 °C with rabbit polyclonal anti-I Kappa B alpha (IKBa, 1:2000, #44D4, Cell Signaling Technology, USA), anti-pIKBa (1:2000, #14D4, Cell Signaling Technology, USA), anti-p65 (1:2000, #3033, Cell Signaling Technology, USA), anti-phospho-p65 (1:2000, #3039, Cell Signaling Technology), p62 (1:2000, PM045, Medical & Biological Laboratories Co., Ltd., Japanese), LC3 (1:2000, L7543, Sigma–Aldrich Co., USA), and mouse polyclonal β-actin antibody (1:5000, A1978, Sigma–Aldrich Co.). After rinsing, the membranes were soaked in blocking buffer with secondary antibodies (purified polyclonal antibody to rabbit or to mouse, 1:200–5000, KPL, USA) for an hour. After rinsing again, the membranes were visualized with the chemiluminescence method (Cat 32106, Pierce™ ECL Western Blotting Substrate, ThermoFisher Scientific, USA).

MiR-30a targets IL1a and regulates islet functions as an inflammation buffer and response factor

Xin Jiang, Chenke Xu, Fan Lei, Liao Meijian, Wei Wang, Naihan Xu, Yaou Zhang, Weidong Xie

Supplemental Table S1 Nucleic acid sequences for miRNAs and siRNA mimics.

Gene names	Sequences (5' to 3')
miR-30a mimics	UGUAAACAUCCUCGACUGGAAG
Negative controls	UUCUCCGAACGUGUCACGUTT
miR-30a inhibitors	CUUCCAGUCGAGGAUGUUUACA
miRNA inhibitor NC	CAGUACUUUUGUGUAGUACAA

MiR-30a targets IL1a and regulates islet functions as an inflammation buffer and response factor

Xin Jiang, Chenke Xu, Fan Lei, Liao Meijian, Wei Wang, Naihan Xu, Yaou Zhang, Weidong Xie

Supplemental Table S2 Primers of mouse IL1a 3′-UTR and its mutated fragments

**Gene names**	**Primers (5' to 3')**
IL1a-3’UTR	Forward: GCCTCTAGATATTTCGGGAGTCTATTC
	Reverse: GCCCTCGAGTAGAGTCTTTTTGATCCTC
IL1a-3’UTR-mut	Forward: TGAAAGCTAAGCCTCTTTGTAAGAGAAGAG
	Reverse: TTTAGAATTACAGAGACTCAGCACA

MiR-30a targets IL1a and regulates islet functions as an inflammation buffer and response factor

Xin Jiang, Chenke Xu, Fan Lei, Liao Meijian, Wei Wang, Naihan Xu, Yaou Zhang, Weidong Xie

Supplemental Table S3 Primers for PCR.

Gene names	NCBI Accession No.	Primers (5' to 3')	Sizes （bp）
Mouse IL1a	NM_010554.4	Forward: TCTGCCATTGACCATCTC<br>Reverse: ATCTTCCCGTTGCTTGAC	182
Mouse IL6	NM_031168	Forward: CTGCAAGAGACTTCCATCCAG<br>Reverse: GTGGTATAGACAGGTCTGTTGG	131
Mouse TNFa	NM_013693.2	Forward: GGGCTTCCAGAACTCCA<br>Reverse: GCTACAGGCTTGTCACTCG	213
Mouse Ins1	NM_008386	Forward: CACTTCCTACCCCTGCTGG<br>Reverse: ACCACAAAGATGCTGTTTGACA	81
Mouse Ins2	NP_001172013.1	Forward: GCTTCTTCTACACACCCATGTC<br>Reverse: AGCACTGATCTACAATGCCAC	147
Mouse		Forward: GCCGCCACCCCAGTTTAC	190

PDX-1	NM_008814.3	Reverse: CCCAGGCTCGGTTCCATT	
Mouse NeouroD1	NM_010894	Forward: GACCCAGAAACTGTCTAAAATAGAGACA<br>Reverse: AAGGAGACCAGATCAGGGCTTT	105
Mouse CREM	NM_0012715 05.1	Forward: GCTGAGGCTGATGAAAAACA<br>Reverse: GCCACACGATTTTCAAGACA	87
Mouse BHLHE22	NM_021560.4	Forward: GGGGAGAGGGAGGTTTAGTG<br>Reverse: CCCTTTCATCACTTGCCAAT	213
Mouse Actin	NM_007393	Forward: GTGACGTTGACATCCGTAAAGA<br>Reverse: GCCGGACTCATCGTACTCC	245
Human IL1a	NM_000575	Forward: ATCATGTAAGCTATGGCCCACT<br>Reverse: CTTCCCGTTGGTTGCTACTAC	131
Human Actin	NM_001101.3	Forward: CATGTACGTTGCTATCCAGGC<br>Reverse: CTCCTTAATGTCACGCACGAT	250
